# A Social Dimension for a New Industrial Strategy for Europe

**DOI:** 10.1007/s10272-021-0969-6

**Published:** 2021-06-04

**Authors:** Cinzia Alcidi, Sara Baiocco, Francesco Corti

**Affiliations:** Centre for European Policy Studies (CEPS), 1 Place du Congrès, 1000 Brussels, Belgium

## Abstract

The impact of the COVID-19 pandemic has not been equal across economic sectors, age groups, education levels and employment status.

The COVID-19 pandemic has completely disrupted the European labour markets. Demand has collapsed in certain sectors, teleworking has become the norm in others, and the use of digital technologies in services and businesses has experienced a significant acceleration. While the use of short-time work schemes contributed – especially in the initial months of the crisis – to preserving employment and avoiding massive layoffs, unemployment and the inactivity rates are increasing and this course is expected to continue. Such a constellation poses significant challenges to European labour markets. Multiple questions arise around job creation and destruction, up- and re-skilling of the labour force, spatial or sectoral relocation of dismissed workers and the quality of the newly created jobs.

In March 2020, the European Commission (2020a) published a communication on the new industrial strategy for Europe. Such a strategy could play an important role in addressing, directly or indirectly, the above-mentioned challenges. It has the potential to affect the development of economic sectors, and hence job creation and destruction, the quality of jobs and the demand for skills; furthermore, it will influence the spatial dimension, the pace and time horizon of industrial change. Against this backdrop, the new EU industrial strategy should not neglect the labour and social dimension. Lacking such a dimension would indeed not only be inconsistent with the principles of cohesion, convergence and social progress defined in the European Pillar of Social Rights, the UN Sustainable Development Goals and the EU Treaties,[1] it would jeopardise the relevance and the legitimacy of a strategy that aspires to have a strong EU connotation.

The present contribution aims to take stock of the main labour market trends, notably zooming into the impact of the coronavirus pandemic, and draw recommendations on how to codify the social dimension of an EU industrial strategy in line with the social objectives of the EU.

## The asymmetric impact of COVID-19

In the first half of 2020, the EU economy plunged into an unprecedented recession. Despite the deep economic contraction, the impact of the coronavirus pandemic on the labour market has been only partially visible through the usual labour market indicators. In October 2020, the EU unemployment rate was about one percentage point higher than the year before. Similarly, employment declined only to a limited extent, compared to the GDP fall,

**Cinzia Alcidi**, Centre for European Policy Studies, Brussels, Belgium.

**Sara Baiocco**, Centre for European Policy Studies, Brussels, Belgium.

**Francesco Corti**, Centre for European Policy Studies, Brussels, Belgium; and University of Milan, Italy.

1 See Articles 151, 153 and 155 of the Treaty on the Functioning of the European Union.

Figure 1

**The impact of the COVID-19 pandemic on employment in the EU**

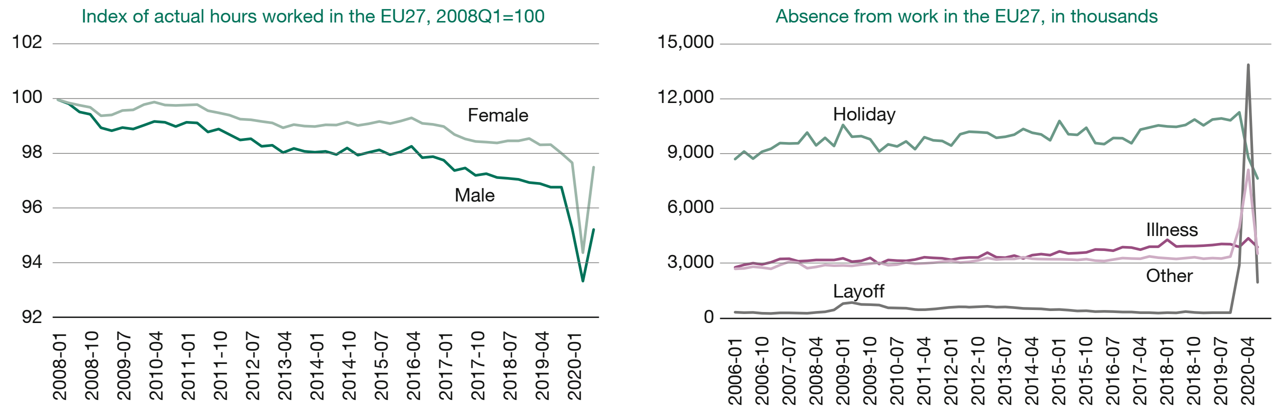

Note: The index represents quarterly changes in the actual hours worked in the main job for full time workers. It indicates the percentage of change in the total actual hours of work in the considered quarter of a year compared to the total actual working hours in 2008.

Source: Authors' own elaboration based on Eurostat.

by less than 1% in the third quarter on a quarter-over-quarter basis, according to Eurostat. The fall is not very different from the one of 2009, but it appears small relative to the magnitude of the drop in GDP, which was far larger in 2020 than in 2009.

The relatively mild impact on employment should not lead to the underestimation of the pandemic's effects on labour markets. Indeed, if the number of unemployed increased by a few thousand, the drop in the active population in the second quarter of 2020 compared to the end of 2019 was around six million people (Eurostat, 2021). Similarly, actual hours worked fell abruptly in the second quarter of 2020; even though the rebound in the third quarter of 2020 has been strong, the level was well below that of late 2019 (see the left-hand side of Figure 1). EU countries also experienced a sharp rise in absences from work, associated with temporary layoffs. In the third quarter of 2020, temporary layoffs were still higher than at the end of 2019 (see the right-hand side of Figure 1).

The impact of the COVID-19 pandemic has not been equal across economic sectors, age groups, education levels and employment status.

Contact-intensive activities (e.g. food and accommodation services, art, entertainment and recreational activities) have been hit particularly hard by the pandemic, both in terms of the fall in employment and actual hours worked (see Figure 2). Workers in these occupations are often female, less-educated, low-paid and young. Among

Figure 2

**Quarter-on-quarter change in employment in the EU27, 2020**

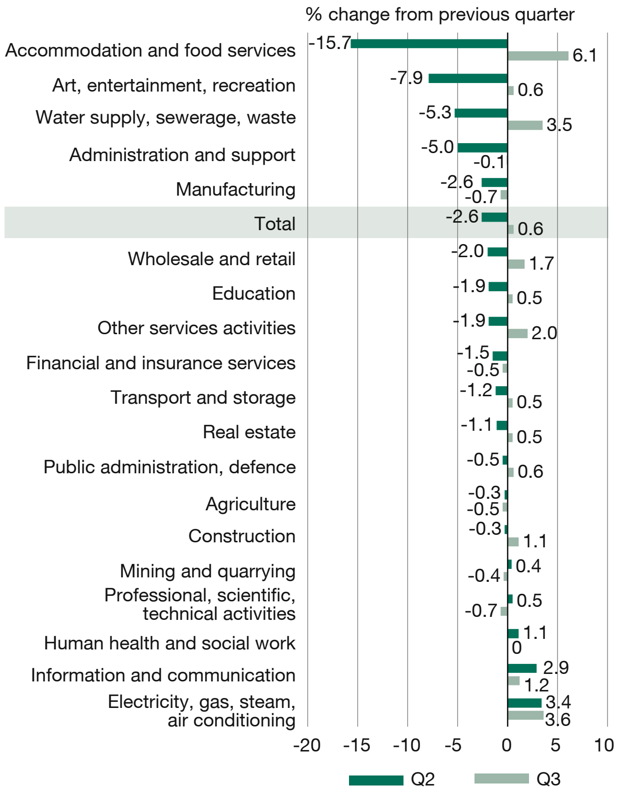

Source: Authors' own elaboration based on Eurostat.

Figure 3

**Employment by education level in the EU27**

percentage of total employment

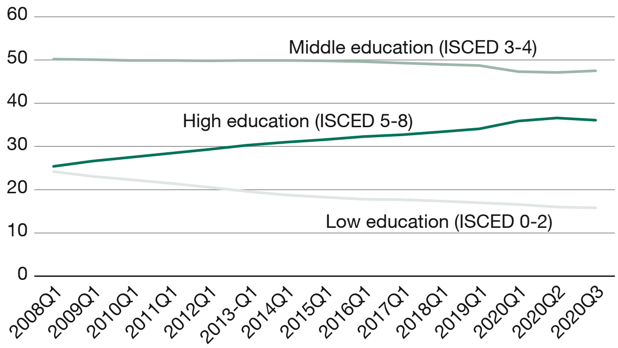

Source: Authors' own elaboration based on Eurostat.

them, the young and the less-educated workers are more at risk of unemployment because they are more likely to be in temporary contracts and less likely to be in teleworkable occupations, respectively. Furthermore, the sectors mostly affected by the containment measures in the second quarter of 2020 have shown only a modest rebound during the third quarter, when the stringency measures were relaxed. This is because their capacity to fully recover to the pre-crisis levels of employment will depend not only on the duration of the containment measures, but also on potential changes in consumption patterns and in the structural features of the production system (e.g. the prevalence of small and medium-sized enterprises that are most exposed to default, automation of production systems or digitalisation of the production processes in a non-reversible way).

Because of its sectoral impact, the COVID-19 pandemic appears to have amplified pre-existing trends in skills demand and employment. Since the beginning of the pandemic, the share of highly educated workers has risen, the share of less-educated workers has fallen and workers with a mid-level education have experienced a milder decrease (see Figure 3).

By accelerating digitalisation and hitting mostly sectors where the level of education of workers tends to be low (e.g. accommodation, food and services), the coronavirus pandemic has accentuated the bifurcation in labour demand by high/low education levels. Based on the experience of the economic crisis post-2010 and the highly uncertain future of some sectors employing less-educated workers, there is a growing concern that employment of less-educated workers will not rebound strongly.

Teleworking represented an important variable in cushioning the impact of the pandemic. Sectors and occupations that could promptly adopt a teleworking regime have experienced a limited impact on employment and hours worked. According to a European Central Bank study (Anderton et al., 2020), in 2019 teleworkable jobs accounted for 33% of employees and 46% of annual earnings in the euro area. This is consistent with the fact that remote working is more prevalent in highly paid jobs. At the aggregate level, EU countries exhibit substantial differences in the share of potentially teleworkable jobs, essentially linked to the structure of the economy. It varies from above 50% in Belgium, Luxembourg and Sweden to less than 20% in Greece, Romania and Spain. That said, the percentage of people in potentially teleworkable sectors actually reporting to have worked from home either usually or sometimes in 2019 was less than 10%.[2] The large gap between potential and actual take-up rates for teleworking suggests a low degree of preparedness to promptly deploy remote working as a tool to cope with the pandemic in terms of both digital equipment and employment regulation. This explains why the sudden shift to telework in 2020 – even though it contributed significantly to preserving jobs – has been a dramatic change for both workers and companies. The former experienced new working conditions and had to adapt their skills quickly to the new way of working. The latter suddenly had to adapt work organisation. While the effects on wellbeing, productivity and innovation performance are still unclear, this creates the possibility of high potential cost savings that could lead to a permanent shift to telework in the future.[3]

Lastly, the asymmetric impact of COVID-19 on European labour markets appears to be affecting employment income inequalities. According to Eurostat, the EU median employment income (before government compensations) declined by 5% in 2020 (compared to 2019) driven by absence from work and reduced working hours (Eurostat, 2020). In 12 EU countries, the share of people working 80% or less hours was above 20% and was concentrated in a few sectors.[4] Eurostat estimates also suggest that young employees appear to have experienced income loss that was twice as large as older workers, and temporary workers' loss was more than six times that of permanent workers. In half of the EU member states, the income loss is three to six times higher for the low-income group than in the high-income group. Low-income earners are

2 Still, significant regional differences emerge. For instance, 70% of potential teleworkers report working from home in Stockholm while less than 10% of potential teleworkers engage in remote working in Italy (Anderton et al., 2020).

3 See for instance Frey (2021) and Eurofound (2021a).

4 Food and accommodation registered losses of almost 20%.

also more likely to have their employment income reduced because of unemployment transitions rather than reduced working hours or absence from work.

One of the reasons behind the significant employment income loss of low-paid and young workers is related to the type of employment contract. Low-paid jobs are traditionally regulated by non-standard employment contracts (fixed and short-term contracts) or fall under a self-employment regime, which usually does not allow access to traditional social protection schemes. As stressed by Spasova et al. (2021), these workers have only benefitted to a minor extent from the social protection measures put in place during the pandemic (e.g. short-time work schemes, but also sick pay and sickness benefits) or remained excluded.[5] Many of the measures adopted are temporary (sometimes one-off), flat rate and means-tested benefits.[6] Overall, even though temporary adjustments to social protection schemes have been introduced to respond to the COVID-19 crisis, these have largely benefitted workers who had formal access to social protection. This aspect is important in a forward-looking perspective because these types of contracts may become even more widespread than in the past during economic recovery.

### The link between COVID-19 and the digital transition

The pandemic crisis erupted at a time when major long-term transformations are shaping the European labour markets. As observed above, COVID-19 is accelerating digitalisation by forcing an upgrade in digital skills for the development and adoption of new digital tools and platforms. It is imposing teleworking, when possible, in a way that it is likely to stay. For non-teleworkable economic activities, an accelerating of automation of production processes is considered likely to happen (Pouliakas, 2018; Chernoff and Warman, 2021).

These trends may further disrupt European labour markets by deepening sectoral and territorial asymmetries caused by the already ongoing digitalisation processes. For instance, in the case of automation, Pouliakas (2018) estimates that future job openings in the two largest subsectors of manufacturing will be relatively low. How fast automation happens depends on the capacity and willingness of companies to invest in new technology as well as the territorial economic structure. A recent Joint Research Centre study (Arregui Pabollet et al., 2019) shows that job losses due to automation are likely to be higher in southern and central European countries, while Nordic countries seem to face a lower risk.[7] Depending on the degree of industrial specialisation, the impact of automation and digitalisation is likely to vary even across regions within the same country. Regions with higher innovation capacity tend to have more high-paid, in-demand jobs, while regions with a lower innovation capacity tend to have more low-paid jobs at higher risk of automation.

### The objectives of a new industrial strategy centred on quality jobs

Bringing a social dimension in the new industrial strategy for Europe entails addressing two broad main challenges. The first challenge concerns skills and more specifically the transferability of skills from jobs destructed to jobs created as well as the upgrade and adaptation of skills to respond to changing job tasks following the digital transition and its acceleration induced by the coronavirus crisis. The second challenge concerns the quality of the new jobs created. The COVID-19 pandemic has highlighted the vulnerability of workers in low-quality jobs, lacking any safety net in the face of shocks, and new challenges in working conditions. The increasing resort to non-standard employment arrangements raises concerns about developments in wages, job security and working conditions.

To credibly address such challenges, a truly European industrial strategy cannot be devised in isolation from other EU objectives and policies. As observed above, COVID-19 and the digital transition are likely to have an asymmetric regional impact, possibly exacerbating disparities between prospering and declining regions. While it is not for the EU industrial strategy alone to address such an issue, it is crucial from an EU legitimacy perspective that the new EU industrial strategy is coherent and develops synergies with other EU policies and initiatives, especially the EU cohesion policy.

Similarly, member states cannot credibly subscribe to the EU industrial strategy without linking its implementation to national policies, and notably social protection and education policies. Both areas are critical for a modern and sustainable industrial development. To this end, the EU industrial strategy will have to be seconded by an EU coordinated action that aims, firstly, to develop national social

5 According to Eurofound (2021b), in 2020, almost 50% of self-employed interviewed reported that their household had difficulties making ends meet and 73% considered the social protection available is inadequate. Job insecurity appeared to hit young people and women particularly harshly.

6 In nearly all cases, these benefits are being paid by the state budget instead of social security contributions.

7 For instance, less than 50% of non-managerial, professional and technical occupations in the French textile and leather sector could potentially be automated by 2030, whereas in Poland this figure is close to 78%.

Figure 4
**The social dimension of the EU industrial strategy**

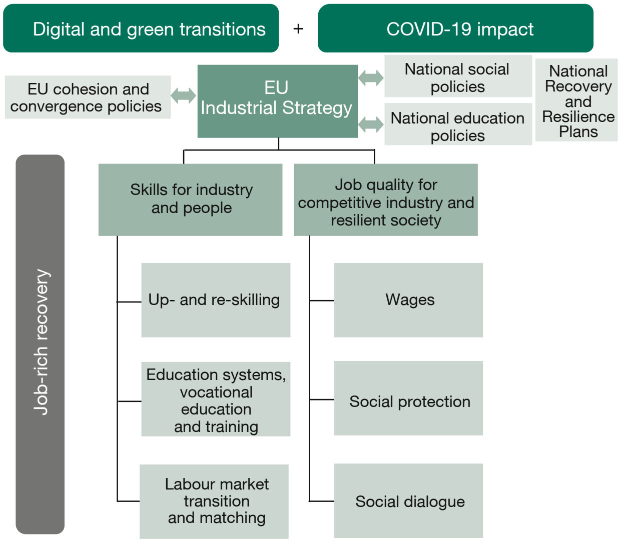

Source: Authors' elaboration.

protection systems to support those who will not be able to thrive in the transition to a sustainable and digital EU industry and may be left outside the labour market for long periods or even permanently. Secondly, coordination will be needed to transform European education and training systems in the member states to ensure the continuous supply of relevant skills and keep enhancing the competitiveness of a transformed industry. The Recovery and Resilience Facility will be the framework in which such a coordinated approach will be guaranteed and monitored. For this, it is important that the National Resilience and Recovery Plans take into consideration the objectives and measures that will define the national implementation of the EU industrial strategy. Figure 4 illustrates the linkages between all these different dimensions and the areas of policy intervention.

## Policies for an EU industrial strategy with a social dimension

With this background in mind, ten policy recommendations focussing on skills and job quality can help to define an EU industrial strategy with a relevant social dimension.

### Skills for industry and people

A key objective of the EU industrial policy should be to develop skills. The EU industrial strategy should aim at enhancing and complementing the skill sets that can support individuals in labour market transitions and ease skills transfers from declining to expanding sectors and occupations, as well as across regions. Enhanced and adjusted skill sets would allow workers to take up the new job opportunities that will be created and industry to count on a talented and skilled workforce during the transitions. Skills development will also support workers that stay in the same job, sector or occupation to upgrade their skills to run new tasks, addressing changing industry's skills needs in a timely manner. Ultimately, having skills among the priorities of an EU industrial policy means recognising the crucial role of human capital for the EU industry to thrive, as well as endorsing people to benefit from industrial development. The *European Skills Agenda*, published in July 2020, provides a key reference framework for the EU industrial strategy to develop policies in this area. To ensure coherence and develop synergies in EU action, it is important that the EU industrial strategy contribute to streamlining the policy discussion, measures and funds in the area of skills under the Skills Agenda's overarching umbrella. In line with actions foreseen by the Skills Agenda and in addition to them, five policy actions should be envisaged.

*Foster up-to-date and future-proof education and training*. EU industrial strategy should foster and create opportunities for industry-led foresight studies to link potential industrial scenarios to educational needs and trends. Such studies, together with constant and refined skills intelligence, should provide a compass on possible industrial developments in the next future and orient the transformation of European education and training systems.

*Incentivise firm-oriented training*. The EU industrial strategy can and should make sure that the firms leading the digital and green transition of the EU industry invest in human capital, and not only in technology or infrastructure. To this aim, it should use financial provisions to incentivise trainings in expanding and strategic sectors. Trainings should help firms to provide continuous upskilling to their employees and initial training (e.g. apprenticeships, work-based learning) to newcomers.

*Support the establishment of individual-based entitlement to adult education and training*. To support access to adult education and training opportunities and the acquisition of skills to transit across sectors and occupations, the EU industrial strategy should endorse the establishment of universal, modular entitlement to incentives for individuals to participate in education and training. This could prove especially useful for individuals managing their own labour market transitions and seeking new job opportunities in order to secure their career paths in the long term.

*Certify industry-relevant skills and make them visible*. The EU industrial strategy should favour the refinement of a transparent and detailed taxonomy of skills relevant to industrial development, especially for strategic and expanding sectors, to be used by firms, individuals and intermediary actors in labour market transitions (e.g. social partners, education and training providers, public and private employment services). Such taxonomy could start from what is already available in the European Skills, Competences, Qualifications and Occupations classification and should also be informed by the Industrial Forum and similar networks such as the Blueprint for Sectoral Cooperation on Skills.

*Manage skill-matching and labour market transitions*. The new EU industrial strategy should support the enhancement of public and private employment services by leveraging the potential of digitalised labour market services. Such services should be made available to advise firms when recruiting and to provide training for new jobs. Their role can be crucial to guiding individuals facing labour market transitions and in need of skills assessment and validation as well as up- and re-skilling.

## Job quality for a competitive industry and a resilient society

In order to ensure a competitive industry and a resilient society, a priority of the EU industrial strategy must be the promotion of job quality. Job quality is a multidimensional concept, which includes characteristics of work and employment that have been proven to have a causal relationship with workers' health and well-being. In the context of an EU industrial strategy, the concept of job quality is linked to three objectives related to its measurable dimensions: wages, social protection and social dialogue.[8] Fair wages, social protection and social dialogue figure as prominent features of the European social model. The European Pillar of Social Rights explicitly acknowledges that all workers have the right to fair wages that provide for a decent standard of living and adequate social protection, regardless of the type and duration of their employment relationship. In addition, the Pillar recognises the importance of social dialogue and the necessity for social partners to be consulted, to negotiate and to conclude collective agreements in matters relevant to them. Five main policy recommendations can make this happen.

*Ensure fair minimum wages*. A new European industrial strategy that aims for competitiveness and social fairness should be accompanied by an initiative to ensure fair wages across the EU. The recently proposed EU Directive on adequate minimum wages (European Commission, 2020b) could help achieving such objective. It would play an important role in preventing in-work poverty and stimulate legal employment. At the same time, by ensuring compulsory implementation of fair minimum wages in a coordinated manner across the whole of the EU, it would create a level playing field and promote wage convergence, in particular between countries in Central and Eastern Europe and Western Europe.

*Promote social dialogue*. The new EU industrial strategy should ensure an inclusive governance by involving social partners in co-designing and co-creating its actions. Social dialogue, and notably collective bargaining, is a key means through which employers' organisations and trade unions can establish fair wages and working conditions. The decline in collective bargaining coverage has been associated with a downward pressure on wages, a high share of low-wage earners in a number of countries and increasing wage inequality. To guarantee that the industrial transition goes hand in hand with job quality, the EU industrial strategy should ensure effective social dialogue and guarantee workers' information and participation in the transition and restructuring processes.

*Encourage socially sustainable business models*. A forward-looking EU industrial strategy should aim to move away from a social protection system that hinges on a person's employment status and towards social protection that is neutral with regards to the forms of employment and self-employment. It should encourage a European benchmark for industrial business models that embed a new balance of the (production and market) risks borne by firms and workers. This entails ensuring neutral social protection against unemployment, sickness and other life circumstances independently of employment status (European Commission, 2019).

*Monitor, assess and evaluate job quality*. In order to adequately monitor the implementation of the new EU industrial strategy, the Commission will propose the introduction of Key Performance Indicators (KPIs) that measure the transformation of European industry and its resilience in the aftermath of the pandemic. Job quality indicators should be included in the list of KPIs. They should be coherent with the revisited Social Scoreboard within the European Semester and the new Recovery and Resilience Scoreboard. KPIs should include indicators of employment insecurity and job status insecurity, social dialogue, working time and work-life balance, autonomy, work intensity and work relationships.

8 The OECD, the ILO and Eurofound have a framework defining job quality. Here we focus on few dimensions we believe are relevant in the context of the EU industrial strategy.

*Manage the transition towards structural telework*. Teleworking is expected to be used in a much larger and persistent way, but its effects are still highly uncertain. Monitoring and analysing its implications on total and sectoral employment, employment relations, working conditions, innovation and productivity should be a key priority for the new EU industrial strategy. A structural shift to telework could be informed by defined pilot programmes to understand the transformation of working environments in a blended regime of remote and office work (e.g. hot desk, open spaces) before deploying these regimes on a large scale. Social partners can play a key role in managing such a shift, as well as in better understanding its implications.

## Concluding remarks

While the pandemic is not yet under control, national governments are shifting their agendas from the emergency response to the long-term recovery. The new EU industrial strategy can play a key role in making the recovery sustainable and inclusive and contribute to shaping a new EU economic and social model. A truly European industrial strategy that in addition to fostering competitiveness of companies supports policies that foster people's skills and preserve job quality will enjoy a wider legitimacy, and hence foster EU legitimacy.
